# Hyperhaploid plasma cell myeloma characterized by poor outcome and monosomy 17 with frequently co-occurring *TP53* mutations

**DOI:** 10.1038/s41408-019-0182-z

**Published:** 2019-02-19

**Authors:** Jess F. Peterson, Ross A. Rowsey, Cherisse A. Marcou, Kathryn E. Pearce, Cynthia M. Williamson, Lori A. Frederick, Patricia T. Greipp, Rhett P. Ketterling, Shaji Kumar, David S. Viswanatha, Mei-Yin Polley, James M. Fink, Kaaren K. Reichard, Daniel L. Van Dyke, Linda B. Baughn

**Affiliations:** 1 Division of Laboratory Genetics and Genomics, Department of Laboratory Medicine and Pathology, Mayo Clinic, Rochester, MN USA; 2 Division of Hematopathology, Department of Laboratory Medicine and Pathology, Mayo Clinic, Rochester, MN USA; 3 Division of Hematology, Department of Internal Medicine, Mayo Clinic, Rochester, MN USA; 4 Division of Biomedical Statistics and Informatics, Department of Health Sciences Research, Mayo Clinic, Rochester, MN USA; 5 Department of Pathology, Hennepin Healthcare, Minneapolis, MN USA

Plasma cell myeloma (PCM) is a clonal PC neoplasm that represents the second most common and mostly incurable hematopoietic malignancy comprising ~20% of all hematologic-related cancer deaths in the United States^1,2^. The diagnosis of PCM requires evidence of clonal expansion of PCs representing ≥10% of the bone marrow, elevated monoclonal protein concentration in the serum and/or urine, lytic bone lesions, and/or end organ damage^3–5^. Multiple risk stratification systems and prognostic-based therapeutic approaches have been devised incorporating a variety of clinical metrics including host factors such as age, performance status, comorbidities, serum beta-2 microglobulin, albumin, lactate dehydrogenase, and proliferation indices.

Cytogenetic analysis plays a critical role in risk stratification of patients with newly diagnosed PCM and subsequent disease progression^3–5^. Recurrent structural and numeric chromosomal abnormalities identified by fluorescence in situ hybridization (FISH) stratifies patients into standard- or high-risk groups that mainly estimate overall survival (OS)^3–5^. High-risk cytogenetic abnormalities include immunoglobulin heavy chain (IGH) translocations; t(4;14) (*IGH/FGFR3*), t(14;16) (*IGH/MAF*), t(14;20) (*IGH/MAFB*),17p (*TP53*) deletions, 1p deletions, and 1q duplications. Standard-risk abnormalities include hyperdiploidy (47–57 chromosomes) often involving gains of odd-numbered chromosomes and *IGH* translocations including t(11;14) (*IGH/CCND1*) and t(6;14) (*IGH/CCND3*)^3,4^. Beyond cytogenetic studies, next-generation sequencing (NGS) technologies have significantly expanded the ability to characterize the mutational landscape of PCM, thus providing additional genetic information of clinical value^3,6^.

Hyperhaploidy (24–34 chromosomes), a subtype of low hypodiploidy, is a unique cytogenetic subgroup in PCM that has rarely been described in the literature^7–12^. A recent retrospective clinical series of 33 patients with hyperhaploid PCM demonstrated a poor prognosis with a 5-year survival rate of 23%^10^. Curiously, the same set of odd-numbered disomies typically observed in hyperhaploid clones (chromosomes 3, 5, 7, 9, 11, 15, 18, 19, and 21) are the same set of odd-numbered trisomies typically observed in hyperdiploid PCM, with gains of chromosome 18 observed less than the odd-numbered trisomies^7–13^. Considering that certain monosomies in hyperhaploid clones are associated with high-risk abnormalities, including monosomies 1 (loss of 1p) and 17 (*TP53* deletion), this may contribute to the unfavorable prognosis associated with this cytogenetic subgroup. However, additional likely contributing elements including mutation evaluation and morphologic description of hyperhaploid PCM have not been analyzed.

To further characterize this rare cytogenetic subgroup, we describe 22 cases of hyperhaploid PCM utilizing conventional chromosome, FISH and NGS studies along with morphologic and survivorship data (See supplementary materials and methods). To achieve this goal, we conducted a 10-year retrospective review of the Mayo Clinic cytogenetic database (2005–2015) following Institutional Review Board approval to identify hyperhaploid PCM karyotypes with chromosome modal numbers between 24 and 34 chromosomes, in addition to karyotypes with chromosome modal numbers between 48 and 68 that may represent a doubled hyperhaploid clone. Doubled hyperhaploid clones (48–68 chromosomes) in the absence of a hyperhaploid clone (24–34 chromosomes) were required to have supporting FISH evidence of hyperhaploidy. Karyotypes that included more than two marker chromosomes of unknown origin described in the stemline of the karyotype were excluded.

We identified 18 hyperhaploid cases analyzed by the Mayo Clinic Genomics Laboratory plus four cases from Hennepin Healthcare (HHC) Cytogenetics Laboratory, yielding a total of 22 hyperhaploid PCM cases (Table 1) comprised of 11 male and 11 female patients (M:F ratio, 1:1). Age at the time of first abnormal cytogenetic analysis (median age = 54 years; range: 39–80 years) was significantly lower compared to the age of a non-hyperhaploid cohort of patients with first abnormal chromosome and PCM FISH results collected during the same timeframe (2005–2015) (median age = 65 years; range: 28–87 years) (*p-value* = 0.001; Supplementary Table 1 and materials and methods). All patients were deceased at time of censorship with only one patient in our data survived beyond 4 years. Bone marrow aspirate and core biopsy morphology, light-chain status, and plasma cell labeling index (PCLI) or S-phase characteristics from eight patients are presented in Supplementary Table 2.

**Table 1:** Patient characteristics of hyperhaploid PCM cohort

Case	Site	Sex	Age (years)	Karyotype	FISH results	TP53 mutation	Time to death (months)
1	Mayo	F	39	33,X,add(1)(p11), +3, +del(5)(q13q35), +add(6)(p23), +add(7)(p22),add(8)(q22), +9, +11, +14, +15, +16, +18,add(20)(q11.2)[1]/46,XX[29]	Monosomies 13, 17; three copies of *CCND1*	NT	41.3
2	Mayo	M	54	34,X,dup(1)(q21q32), +del(3)(q12), +5, +7, +8, +9, +11, +14, +15, +18, +19, +21[1]/54~69,idemx2[cp18]/46,XY[1]	Monosomies 13, 17; 5’*IGH* deletion; near-tetraploid	p.Arg196*	4
3	Mayo	M	71	32,X, +3, +5, +7, +9, +add(10)(q24), +15, +18, +22, +mar[1]/46,XY[19]	Monosomies 13, 14, 17	NT	12.9
4	HHC	F	61	60~62,XX, +3, +3, +6, +7, +7, +8, +9, +9, +11, +11, +11, +15, +15, +16,−18,−18, +19, +19, +20, +2mar[cp2]/46,XX[18]	Monosomies 1, 8, 13, 14, 17	NT	1.4
5	Mayo	M	49	31~32,del(X)(q13), +add(3)(p21), +add(7)(p22), +add(9)(q11), +11, +15,−18, +21, +2mar[cp14]/ 61~62,idemx2, +2mar[cp6]	Not performed	NT	0.9
6	Mayo	F	52	30,X, +3, +7, +9, +11, +18, +add(19)(p13.3), +21[3]/51~54,idemx2,−3,−4,−7,−18,−add(19)(p13.3),−22, +2~3mar[cp8]	Not performed	None	30.1
7	Mayo	F	55	32~34,X, +3, +5, +7, +9, +add(11)(p15),add(14)(p11.2), +18, +19, +21, +r, +1~2mar[cp7]/66,idemx2[1]/46,XX[12]	Monosomies 13, 14, 15, 17	None	25.8
8	Mayo	F	64	31,X, +3, +7, +9, +11, +add(15)(p11.1),add(16)(q12.1), +18, +19, +21[10]/46,XX[10]	Monosomies 13, 14, 15, 17; near-tetraploid	p.Phe270Ser	11.2
9	Mayo	F	49	33,X, +3, +7, +9, +10, +11, +15, +18, +19, +21, +mar[3]/XX[15]	Not performed	NT	9.9
10	Mayo	M	56	31,X,add(1)(q32), +add(3)(q21), +add(5)(q11.2), +7, +9, +11, +15, +18, +del(22)(q13)[19]/46,XY[1]	Not performed	NT	4.2
11	Mayo	M	50	32,X, +3, +5, +7, +9, +11, +15,der(16)t(16;17)(q13;q11.2), +18, +19, +21[5]/46,XY[15]	Monosomies 13, 14, 17	None	4.4
12	Mayo	F	72	33,X, +1, +2, +6, +7, +11, +14, +18, +19, +21, +22[6]/46,XX[7]	Not performed	p.Arg156Pro	75.5
13	Mayo	M	50	31~33,XY, +3, +5, +7, +add(9)(p13), +11, +15, +18, +19, +mar[cp7]/46,XY[3]	Monosomies 13, 14, 17; trisomy 9	NT	6.8
14	Mayo	M	44	34,X, +3, +5, +add(7p22), +9, +10, +11, +15, +18, +19, +add(21)(q22), +mar[6]/46,XY[14]	Monosomies 13, 14, 17	None	45.8
15	Mayo	M	43	34,X, +3, +5, +7, +8, +9, +9, +11, +15, +18, +19, +21[2]/46,XY[28]	Monosomies 13, 14, 17	p.Asp48Alafs*5	NA
16	Mayo	F	80	32,X, +3, +5, +7, +9, +11, +15, +add(15)(p11.2), +19, +21[5]/46,XX[12]	Monosomies 13, 14, 17	p.Pro250Thr	18
17	Mayo	F	53	33,X,der(1;21)(q10;q10), +3, +5, +7, +9, +11, +add(14)(q32), +15, +18, +19, +21[4]/46,XX[16]	Monosomies 4, 6, 13, 16, 17, 20; *IGH/MYC* fusion	p.Thr253Pro	12.4
18	Mayo	F	71	30,X, +3, +5, +7, +9, +add(11)(q25), +15, +add(19)(q13.3),−21, +1~2mar[cp3]/46,XX[20]	Monosomies 8, 13, 17, 20	p.Tyr220Metfs*27	22.4
19	HHC	F	54	32,X, +der(3)t(2;3)(q33;q29),der(4)inv(4)(p16q13)ins(4;?)(p16;?), +5, +7, +8, +9, +11, +15, +19, +21[3]/46,XX[17]	Monosomies 1, 4, 13, 14, 16, 17	NT	NA
20	HHC	M	53	34,X,del(1)(q11),ins(1;?)(q21;?),add(1)(q32), +der(3)add(3)(p23)add(3)(q22), +add(3)(q22), +5, +7, +8, +9, +11, +add(11)(q23), +14, +add(14)(p11.1), +15, +18, +19, +21[cp20]/46,XY[1]	Not performed	NT	NA
21	HHC	M	58	30,X, +2, +3, +8, +11, +15, +18, +19,−22, +mar[4]/46,XY[3]	Monosomies 1, 4, 13, 14, 16, 17	NT	NA
22	HHC	M	50	33,X, +3, +5, +7, +9, +10, +11, +15, +18, +19, +21[5]/46,XY[15]	Monosomies 1, 4, 13, 14, 16, 17	NT	NA

Mayo, cases from Mayo Clinic Cohort; HHC, cases from Hennepin Health Care

*NT* not tested; *NA* Time to death information not available for HHC cases

Eighteen cases had at least two hyperhaploid metaphases and met criteria for an abnormal clone for this study (Table 1). Of four cases that did not meet these criteria, three cases (patients 1, 2, and 3) had a single hyperhaploid metaphase cell accompanied by supporting FISH results, and 1 case (patient 4, Supplementary Figure 1) had two metaphases (60–62 chromosomes) representing an apparent doubled hyperhaploid clone supported by FISH results. Six cases (patients 2, 4, 5, 6, 7, and 8) had chromosomal or FISH evidence of both a hyperhaploid and doubled hyperhaploid clone (Fig. 1a–f).

**Fig. 1:**
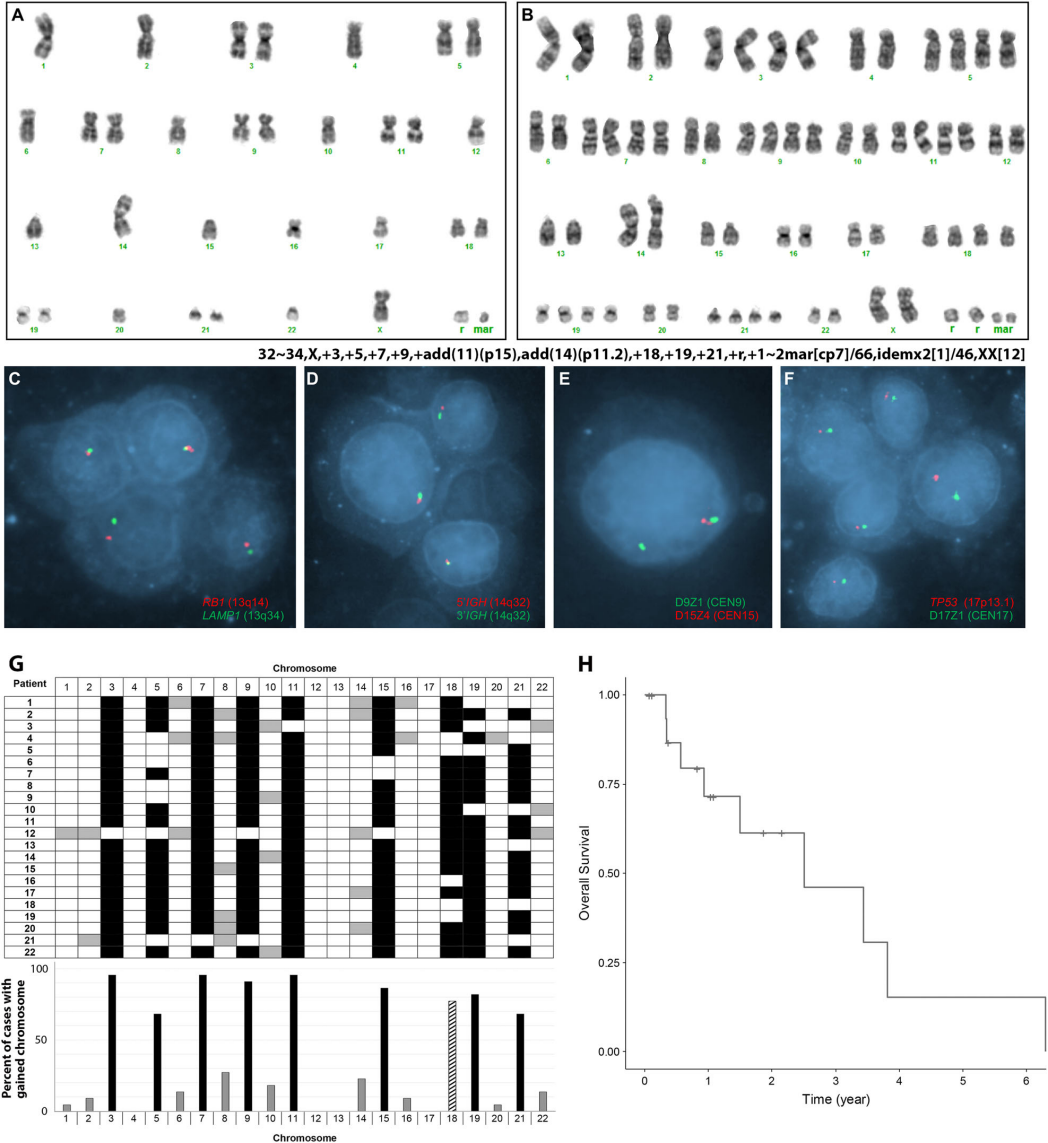
**a** Karyograms from patient 7 with a hyperhaploid and **b** doubled hyperhaploid clones with supporting FISH results in **c**–**f**. **g** Depiction of the gained chromosomes (in reference to a haploid clone) from the cohort of 22 patients with hyperhaploid PCM in both grid format per patient (top) and overall percentage of cases with the gained chromosome (bottom). The most commonly gained chromosomes are indicated by a black bar, less commonly gained chromosomes by a gray bar and chromosome 18 indicated by a pattern bar in graph. Overall survival (OS) was calculated from time of diagnosis to date of death for patients seen at Mayo Clinic (**h**)

The chromosome complement in 21 of 22 cases (excluding patient 4 with only a doubled hyperhaploid clone) ranged from 30 to 34 (mean 32, median 32). The most frequently observed chromosomal gains compared to a haploid chromosome complement (23,X) were chromosomes 3 (*n* = 21; 95%), 11, (*n* = 21; 95%), 7 (*n* = 21; 95%), 9 (*n* = 20; 91%), 15 (*n* = 19; 86%), 19 (*n* = 18; 82%), 18 (*n* = 17; 77%), 21 (*n* = 15; 68%), 5 (*n* = 15; 68%), 8 (*n* = 6; 27%), 14 (*n* = 5; 23%), 10 (*n* = 4; 18%), 22 (*n* = 3; 14%), 6 (*n* = 3; 14%), 16 (*n* = 2; 9%), 2 (*n* = 2; 9%), 1 (*n* = 1; 5%), and 20 (*n* = 1; 5%) (Fig. 1g). No gains of chromosomes 4, 12, 13, or 17 were observed in any of the 22 cases.

Sixteen of 22 cases had concurrent plasma cell FISH studies performed, all of which demonstrated monosomies 13 and 17. In addition to FISH signal patterns suggestive of hyperhaploidy, patient 17 had an *IGH*/*MYC* fusion, and patient 2 had a 5’*IGH* deletion without an *IGH* translocation to the common PCM translocation partners *CCND1*, *CCND3*, *MAF*, *MAFB*, and *FGFR3*. Eleven of 22 cases had subsequent NGS testing performed and revealed a mutation within the *TP53* gene in seven (64%) (patients 2, 8, 12, 15, 16, 17, and 18) (Table 1 and supplementary table 3). Patient 18 also exhibited pathogenic or likely pathogenic mutations in *CDKN1B* and *RB1*. Several patients had co-existing *TP53* mutations and mutations in *TGFBR2* gene (patient 15), *KRAS* (patient 17), or *NRAS* (patient 2). For 17 cases with survivorship data, the median survival was 2.51 years, with an estimated 3-year survival of 46% (95% CI, 22–95%) (Fig. 1h).

Hyperhaploidy in PCM is a rare, high-risk cytogenetic subgroup that exhibits the same set of odd-numbered chromosome gains (in reference to a haploid clone) as in hyperdiploid PCM with the exception of chromosome 18^7–13^. The poor prognosis of hyperhaploid PCM underscores the importance of correctly identifying this cytogenetic subgroup.

Of the 61 total cases of hyperhaploid PCM reported in the literature (including the current study), 39 (64%) had evidence of only a hyperhaploid clone, 19 (31%) had evidence of both hyperhaploid and a doubled hyperhaploid clone, and 3 cases (5%) only had evidence of a doubled hyperhaploid clone^7–12^. However, this latter subgroup may be under-recognized and erroneously misclassified as hyperdiploid without careful evaluation of the apparent chromosome gains and correlative FISH studies. Detection of recurrent structural chromosomal abnormalities in PCM currently relies heavily upon locus- and centromere-specific FISH probes and according to the 2017 revised WHO Classification of Tumours of Haematopoietic and Lymphoid Tissues, the minimal PCM FISH panel should include probes targeting t(4;14) (*IGH/FGFR3*), t(14;16) (*IGH/MAF*), and *TP53*^14^. While using this minimal FISH panel could detect commonly observed monosomies in hyperhaploidy including 4, 14, 16, and 17, doubling of a hyperhaploid clone in the absence of a hyperhaploid clone could be incorrectly interpreted as a “normal” result without a more thorough FISH evaluation. Considering that hyperhaploidy shares the same chromosome gains as hyperdiploidy, centromere-specific probes for chromosomes 3, 7, 9, and 15 should be included in the standard PCM FISH panel in order to not miss the opportunity to discriminate this high-risk cytogenetic subtype. However, newer methodologies to comprehensively evaluate PCM clones, including SNP arrays or NGS assays, will likely eliminate the need for large FISH panels.

Mutations in *TP53* are rarely detected at disease presentation and have been reported in ~3% of newly diagnosed PCMs often in the context of 17p deletions. *TP53* mutations have been reported to increase in frequency with disease progression and are associated with inferior clinical outcome^15,16^. Given 64% of our hyperhaploid cohort (*n* = 11) had pathogenic or likely pathogenic mutations in *TP53* suggests that *TP53* mutation may at least in part account for the poor overall survival of patients with hyperhaploid PCM.

As a large reference genomics laboratory that is unable to routinely obtain complete patient histories, a limitation in this study is our inability to ascertain the exact date of PCM diagnosis for patients not treated at our institution. Therefore, patient survival (as established from the date of the abnormal chromosome study) may be underestimated. The limited sample size of this study (22 patients) also limits the power of the survival estimates. Nevertheless, given that the 4-year survival for PCM exceeds 80% for those patients eligible for ASCT^17^, the survival of patients with hyperhaploid PCM in contrast to all PCM remains poor.

The frequency of hyperhaploidy in various neoplasms is low (~0.2–0.3% of tumors) and is often hallmarked by a non-random pattern of disomic chromosomes distributed among different malignancies^8^. The biological incentive and mechanism for a clone to undergo a massive loss of genomic material with retention of specific chromosomes is currently unclear, but a selective advantage resulting from altered gene expression contributing from the retained chromosomes is likely contributory. In addition, abnormalities in the spindle apparatus necessary for separation of sister chromatids during cell division resulting in specific non-disjunction patterns may possibly contribute to this phenomenon and also to the generation of hyperdiploidy^8,18^. While hyperhaploid and hyperdiploid PCM share a similar pattern of gained chromosomes, how these two entities are related to one another is unclear. While it is possible that hyperhaploidy represents a subclone of hyperdiploidy, we do not see evidence of both hyperdiploidy and hyperhaploidy together within the same specimen by chromosome or FISH studies (Table 1). Although hyperdiploidy and hyperhaploidy share similarly gained chromosomes, IGH gene rearrangements are rarely observed and retention of chromosome 18 along with *TP53* mutations are commonly observed in hyperhaploidy and likely provide a unique selective advantage to this high-risk genetic subgroup. Taken together, hyperhaploid PCM is associated with multiple monosomies including monosomy 17, frequent *TP53* mutations, younger age at the time of abnormal cytogenetic analysis, and decreased overall survival.

## Supplementary information

Supplementary material.

Supplemental Figure 1
