# Supplementary material for: Hyperhaploid plasma cell myeloma characterized by poor outcome and monosomy 17 with frequently co-occurring TP53 mutations
Source: Blood Cancer J. 2019 Feb 19;9(3):20. doi: 10.1038/s41408-019-0182-z (PMC6381150; doi:10.1038/s41408-019-0182-z)
Supplement: Supplementary file 1 — Supplementary material. [file 41408_2019_182_MOESM1_ESM.docx]

**Supplementary materials and methods**

**Conventional chromosome analysis**

Bone marrow cells were cultured, harvested and banded utilizing standard cytogenetic techniques according to specimen-specific protocols from each institution. When available, 20 metaphases were analyzed and results reported per 2016 International System for Human Cytogenomic Nomenclature (ISCN).

**Fluorescence *in situ* hybridization**

The PCM FISH panel at Mayo Clinic was performed on plasma cells identified by cytoplasmic immunoglobulin (cIg) staining as part of routine clinical testing. Plasma cells were evaluated using the following commercially available probes (Abbott Molecular, Des Plaines, IL) unless otherwise specified: locus-specific [*TP73*/1q22 (laboratory-developed test, LDT), *RB1*/*LAMP1*, *TP53*], centromere-specific (D3Z1/D7Z1, D9Z1/D15Z4, D17Z1), break-apart (BAP) [*IGH* (LDT), *MYC*] and dual-color dual-fusion (D-FISH) [*FGFR3*/*IGH*, *CCND3*/*IGH* (LDT), *CCND1*-XT/*IGH*-XT, *IGH*/*MAF*, *IGH*/*MAFB* (LDT)]. The PCM FISH panel at HCMC was performed on non-plasma cell-enriched cells using the following commercially available FISH probes (Abbott Molecular): locus-specific (*TP73*/1q22, *RB1*/*LAMP1*, *TP53*), centromere-specific (D17Z1), and D-FISH (*FGFR3*/*IGH*, *CCND1*/*IGH*, *IGH*/*MAF*). Specimens were subjected to standard FISH pretreatment, hybridization, and fluorescence microscopy according to specimen-specific protocols from each institution.

**Next-generation sequencing**

DNA was extracted from fixed cell pellet samples using the EZ-1 tissue kit (Qiagen, Hilden, Germany). NGS was performed using a 61-gene panel (Supplementary Table 4) targeting genes commonly mutated in PCM. Approximately 200 ng of genomic DNA sheared to approximately 150 base-pairs was utilized for library preparation by the SureSelect XT library kit (Agilent, Santa Clara, CA) and sequenced on the HiSeq platform (Illumina, San Diego, CA) using 2x101 read lengths. NGS data was processed through a proprietary bioinformatics pipeline (Mayo NGS Workbench) for alignment, base calling and insertion/deletion (indel) detection. Results were confirmed by reviewing the BAM files in Alamut Visual (Interactive Biosoftware, Rouen, France).

**Control cohort**

We performed a 10-year review (2005-2015, same timeframe as the hyperhaploid cohort) of the Mayo Clinic cytogenetic database to identify all PCM cases with an abnormal PC-specific clone by conventional chromosome studies that did not represent a hyperhaploid subtype. Patient age and gender were calculated using this control cohort.

**Statistical Methods**
Comparison of nominal variables between groups was assessed with Fisher’s exact tests. Comparison of continuous variables between groups assessed with the Wilcoxon rank-sum test. Overall survival (OS) was calculated from time of diagnosis to date of death for patients seen at our institution (Mayo Clinic). Because date of diagnosis was not available for patients treated at other institutions, survival for these patients was computed from the date of the abnormal chromosome study; as such, survival time was left-censored for these patients. OS was estimated using the Kaplan-Meier method, taking into account left-censored survival among non-Mayo patients.

**Flow cytometric assessment of plasma cell labeling index**

Plasma cell labeling index (PCLI) or S-phase methods were described in ^1,2^.

**References**

1. Greipp PR, Kumar S. Plasma cell labeling index. *Methods Mol Med* 2005; **113**: 25-35; doi 10.1385/1-59259-916-8:25.

2. Kochuparambil ST, Morice WG, Rajkumar SV, Dispenzieri A, Timm MM, Lacy MQ *et al.* Measurement of the Proliferation of Clonal Plasma Cells By Multiparametric Flow Cytometry Is a Clinically Useful Tool in Multiple Myeloma. *Blood* 2014; **124**(21).

**Supplementary Table 1:** Age and gender distributions

|  | Study Cohort | Controls | p-value |
| --- | --- | --- | --- |
| Gender  Female  Male | 11 (50%)  11 (50%) | 89 (46%)  104 (54%) | 0.82 |
| Age in years  Median (range) | 54 (39-80) | 65 (28-87) | 0.001 |

Overall survival estimates (95% CI):

| 1-year | 2-year | 3-year | 4-year |
| --- | --- | --- | --- |
| 0.53 (0.34, 0.83) | 0.29 (0.14, 0.61) | 0.18 (0.06, 0.49) | 0.06 (0.01, 0.39) |

**Supplementary Table 2.**

**Supplementary Table 3.**

| **Supplementary Table 4.** | |  |  |
| --- | --- | --- | --- |
|  |  |  |  |
| *Gene name* | *Chromosome* | *Transcript or genomic posittion* | *Exon or introns covered* |
| *AKT1* | 14 | NM_001014432.1 | Ex3-Ex15 |
| *AKT2* | 19 | NM_001626.5 | Ex2-Ex14 |
| *AKT3* | 1 | NM_001206729.1 | Ex2-Ex14 |
| *AKT3* | 1 | 243668551-243668636 | Intron 13 |
| *ATM* | 11 | NM_000051.3 | Ex2-Ex63 |
| *B2M* | 15 | NM_004048.2 | Ex1-Ex3 |
| *BIRC2* | 11 | NM_001166.4 | Ex2-Ex9 |
| *BIRC3* | 11 | NM_001165.4 | Ex2-Ex9 |
| *BRAF* | 7 | NM_004333.4 | Ex1-Ex18 |
| *CCND1* | 11 | NM_053056.2 | Ex1-Ex5 |
| *CD38* | 4 | NM_001775.3 | Ex1-Ex8 |
| *CDK4* | 12 | NM_000075.3 | Ex2-Ex8 |
| *CDK7* | 5 | NM_001799.3 | Ex1-Ex12 |
| *CDKN1B* | 12 | NM_004064.4 | Ex1-Ex2 |
| *CDKN2A* | 9 | NM_000077.4 | Ex1-Ex3 |
| *CDKN2A* | 9 | 21994138-21994330 | Intron 0 |
| *CRBN* | 3 | NM_016302.3 | Ex1-Ex11 |
| *CUL4A* | 14 | NM_001008895.2 | Ex2-Ex20 |
| *CUL4B* | X | NM_003588.3 | Ex2-Ex22 |
| *CXCR4* | 2 | NM_003467.2 | Ex1-Ex2 |
| *DIS3* | 13 | NM_014953.4 | Ex1-Ex21 |
| *DIS3* | 13 | 73355427-73355494 | Intron 1 |
| *EGFR* | 7 | NM_005228.3 | Ex1-Ex28 |
| *FAM46C* | 1 | NM_017709.3 | Ex2 |
| *FGFR3* | 4 | NM_001163213.1 | Ex2-Ex18 |
| *FGFR3* | 4 | 1805419-1805563 | Intron 8 |
| *GRB2* | 17 | NM_002086.4 | Ex2-Ex6 |
| *IDH1* | 2 | NM_005896.3 | Ex3- Ex10 |
| *IDH2* | 15 | NM_002168.3 | Ex2-Ex11 |
| *IDH3A* | 15 | NM_005530.2 | Ex2-Ex11 |
| *IFNGR2* | 21 | NM_005534.3 | Ex2-Ex7 |
| *IGF1R* | 15 | NM_000875.4 | Ex1-Ex21 |
| *IKZF1* | 7 | NM_006060.5 | Ex2-Ex8 |
| *IKZF3* | 17 | NM_012481.4 | Ex1-Ex8 |
| *IL6* | 7 | NM_000600.4 | Ex1-Ex5 |
| *IL6R* | 1 | NM_000565.3 | Ex2-Ex10 |
| *IRF4* | 6 | NM_002460.3 | Ex2-Ex9 |
| *JAK2* | 9 | NM_004972.3 | Ex3-Ex25 |
| *KDM6A* | X | NM_021140.2 | Ex1-Ex29 |
| *KDM6A* | X | 44919854-44920009 | Intron 13 |
| *KRAS* | 12 | NM_033360.2 | Ex2-Ex5 |
| *MYC* | 8 | NM_002467.4 | Ex1-Ex3 |
| *MYD88* | 3 | NM_002468.4 | Ex1-Ex5 |
| *NFKB2* | 10 | NM_001077494.3 | Ex2-Ex23 |
| *NR3C1* | 5 | NM_000176.2 | Ex2-Ex9 |
| *NRAS* | 1 | NM_002524.4 | Ex2-Ex5 |
| *NSD2* | 4 | NM_001042424.2 | Ex2-Ex22 |
| *PIK3CA* | 3 | NM_006218.2 | Ex2-Ex21 |
| *PIK3CG* | 7 | NM_001282426.1 | Ex2-Ex11 |
| *PIK3R1* | 5 | NM_181523.2 | Ex2-Ex16 |
| *PIK3R1* | 5 | 67584564-67584579 and 67586557-67586662 | Intron 7a and 7b |
| *PIK3R2* | 19 | NM_005027.3 | Ex2-Ex16 |
| *PIM1* | 6 | NM_001243186.1 | Ex1-Ex6 |
| *PIM2* | X | NM_006875.3 | Ex1-Ex6 |
| *PIM3* | 22 | NM_001001852.3 | Ex1-Ex6 |
| *PSMA1* | 11 | NM_148976.2 | Ex2-Ex11 |
| *PSMB5* | 14 | NM_002797.4 | Ex1-Ex3 |
| *PSMB5* | 14 | 23497038-23496954 | Intron 2 |
| *PSMD1* | 2 | NM_002807.3 | Ex1-Ex24 |
| *PSMG2* | 18 | NM_020232.4 | Ex1-Ex7 |
| *PTPN11* | 12 | NM_002834.3 | Ex2-Ex15 |
| *RB1* | 13 | NM_000321.2 | Ex1-Ex27 |
| *STAT3* | 17 | NM_139276.2 | Ex2-Ex24 |
| *TGFBR2* | 3 | NM_001024847.2 | Ex1-Ex8 |
| *TLR4* | 9 | NM_138554.4 | Ex1-Ex3 |
| *TP53* | 17 | NM_001126113.2 | Ex2-Ex10 |
| *TRAF3* | 14 | NM_145725.2 | Ex3-Ex12 |
| *XBP1* | 22 | NM_001079539.1 | Ex1-Ex6 |

**Supplemental Figure 1.** Karyograms (A) from patient 4 with doubled hyperhaploid clone with supporting FISH results (B-F).
