# Supplementary figures and images for: Hyperhaploid plasma cell myeloma characterized by poor outcome and monosomy 17 with frequently co-occurring TP53 mutations
Source: Blood Cancer J. 2019 Feb 19;9(3):20. doi: 10.1038/s41408-019-0182-z (PMC6381150; doi:10.1038/s41408-019-0182-z)

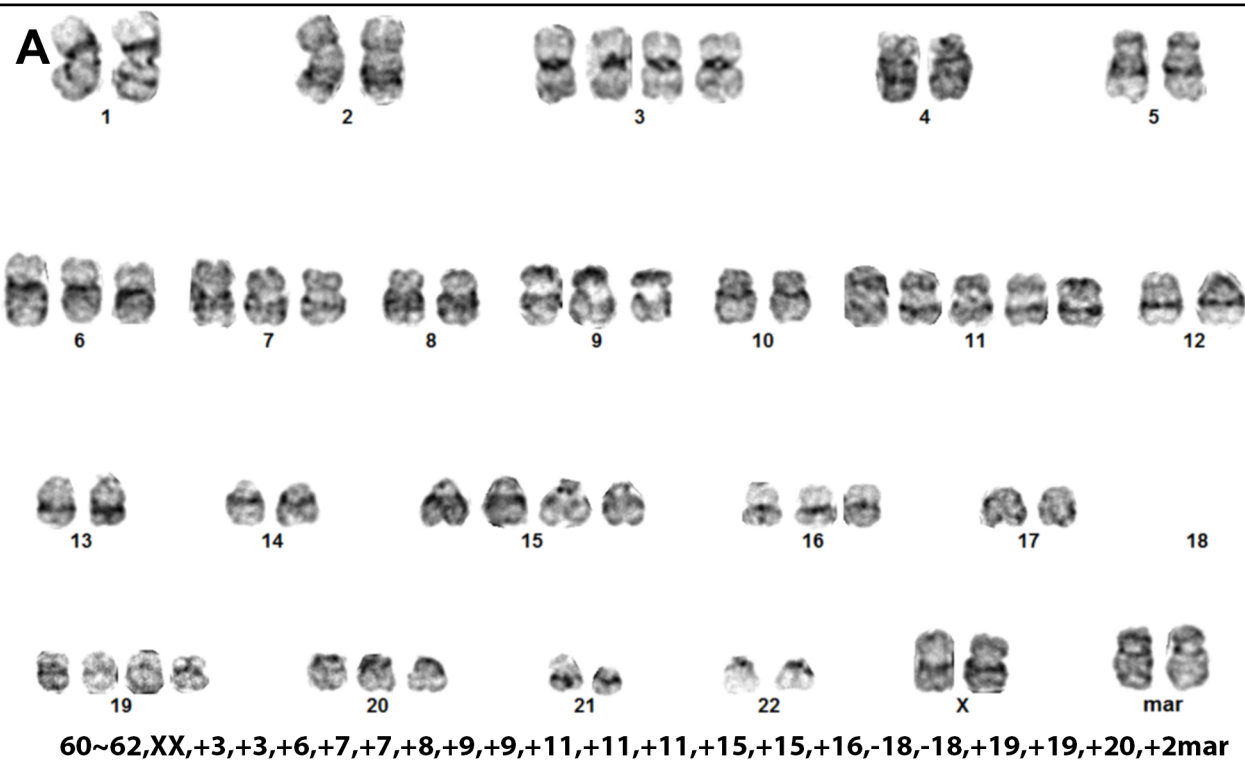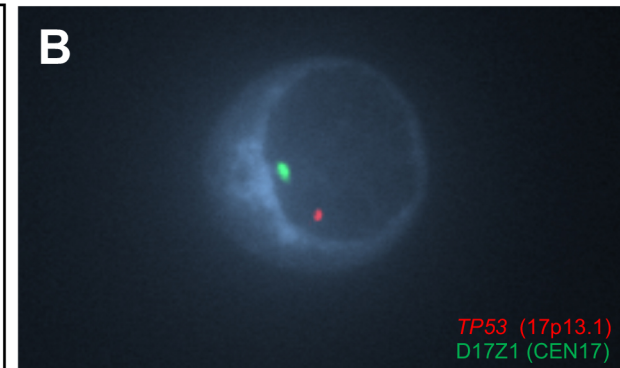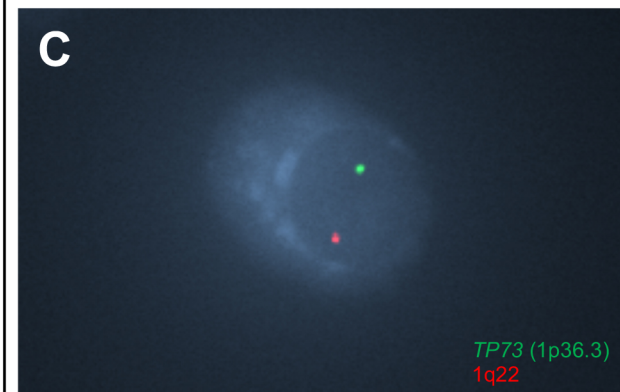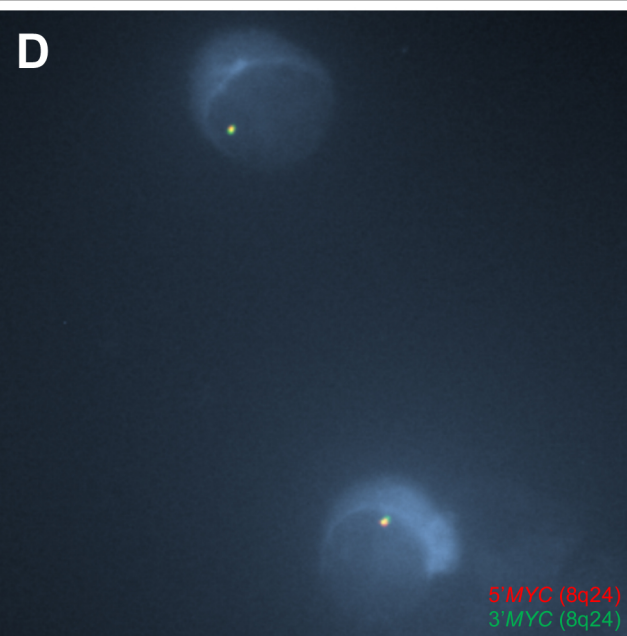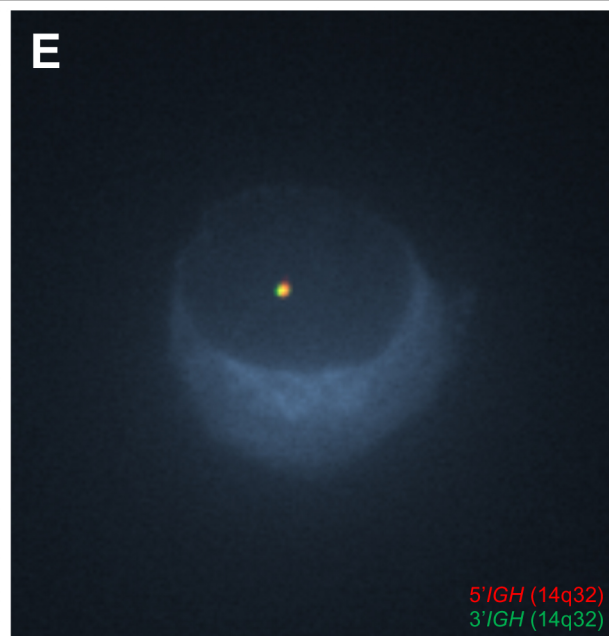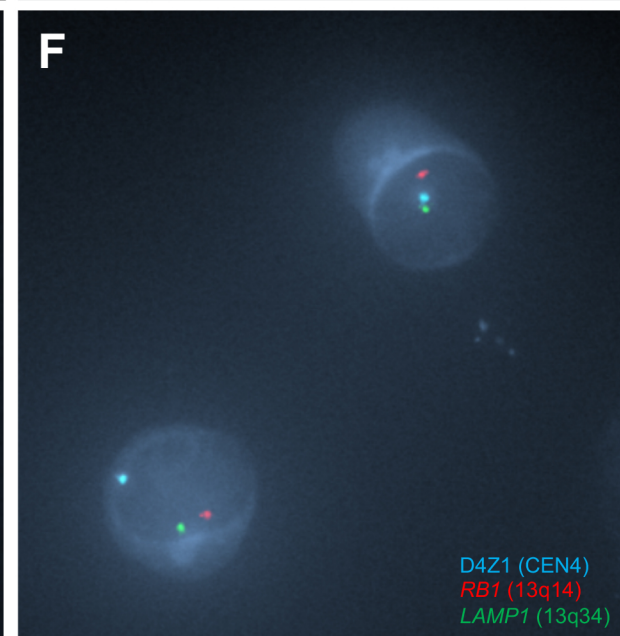

Supplement: Supplementary file 2 — Supplemental Figure 1 [file 41408_2019_182_MOESM2_ESM.pdf]
